# Effects of High Hydrostatic Pressure on *Escherichia coli* Ultrastructure, Membrane Integrity and Molecular Composition as Assessed by FTIR Spectroscopy and Microscopic Imaging Techniques

**DOI:** 10.3390/molecules191221310

**Published:** 2014-12-18

**Authors:** María Prieto-Calvo, Miguel Prieto, Mercedes López, Avelino Alvarez-Ordóñez

**Affiliations:** 1 Institute of Food Science and Technology, University of León, León 24071, Spain; E-Mails: mpriec01@estudiantes.unileon.es (M.-P.C.); miguel.prieto@unileon.es (M.P.); 2 Department of Food Hygiene and Technology, University of León, León 24071, Spain; E-Mail: mmlopf@unileon.es; 3 Teagasc Food Research Centre, Moorepark, Fermoy, Co. Cork, Ireland

**Keywords:** high hydrostatic pressure, *Escherichia coli*, FTIR spectroscopy, cell imaging, inactivation mechanism, food safety

## Abstract

High hydrostatic pressure (HHP) is a novel food processing technology that is considered as an attractive alternative to conventional heat treatments for the preservation of foods, due to its lethal effects on pathogenic and spoilage microorganisms, while causing minor effects on food quality and sensorial attributes. This study is aimed at investigating how HHP treatments at varying intensities in the range 50–900 MPa affect the viability, membrane integrity, ultrastructure and molecular composition of *Escherichia coli*. Results of membrane integrity tests (measurement of cellular leakage and monitoring of propidium iodide uptake through fluorescence microscopy) and ultrastructural observations by transmission electron microscopy demonstrated that HHP gave rise to cellular enlargement, membrane damage or detachment, DNA and protein denaturation and loss of intracellular contents. Fourier-transform infrared (FTIR) spectroscopy analyses evidenced minor changes in molecular composition in response to high pressures, which were mostly observed on the spectral region w_4_ (1200–900 cm^−1^), mainly informative of carbohydrates and polysaccharides of the cell wall. These findings suggest that exposure of *E. coli* cells to HHP causes alterations in their physical integrity while producing minor modifications in biochemical cellular composition. The current study increases the knowledge on the mechanisms of *E. coli* inactivation by HHP and provides valuable information for the design of more effective food preservation regimes based on the integration of mild HHP in combination with other food preservation strategies into a multi-target hurdle technology approach.

## 1. Introduction

High hydrostatic pressure (HHP) is a food processing technology used to maintain the quality attributes of fresh foods, while extending their shelf life through the inactivation of pathogenic and spoilage microorganisms, as well as of endogenous enzymes. In fact, HHP has been proposed as an alternative to thermal processing due to the less detrimental effects it shows on food quality and organoleptic properties [1,2,3].

Great research efforts have been made in recent years to evaluate the safety of HHP-treated foods by studying the inactivation kinetics by HHP of pathogenic microorganisms in food and food models [4]. The foodborne pathogens, *Staphylococcus aureus*, *Listeria monocytogenes*, *Salmonella* spp. and verocytotoxigenic *Escherichia coli* (VTEC), are among the bacterial species most extensively studied with this aim [1]. VTEC, characterized by the production of Shiga toxins, are important foodborne pathogens in the European Union, with 5,671 reported human cases in 2012, which yields a community incidence rate of 1.15 per 100,000 population [5]. Some strains of VTEC are among the most pressure-resistant vegetative cells described to date [6,7]. However, wide variations among *E. coli* strains in HHP resistance have been described, with some strains being inactivated by pressures as low as 200 MPa, whereas others can survive exposures to 600 MPa [6,7,8,9].

Despite much effort in recent years, the main cellular targets and the mechanisms of bacterial killing by HHP have not yet been fully identified [10]. Knowledge on the mechanisms of bacterial inactivation by HHP is, however, essential to define appropriate strategies to guarantee food safety and to optimize process implementation. The cell envelopes have been suggested to be a major target of HHP treatments. Thus, loss of physical integrity of the outer and inner membranes has been shown to occur by means of the increased uptake of fluorescent probes that do not penetrate intact envelopes, the lack of osmotic responsiveness or the loss of intracellular material [11,12,13,14]. Nevertheless, other cellular components, such as ribosomes and cytoplasmic and membrane proteins have been also shown to be affected by HHP treatments [15,16,17,18], and protein denaturation and induction of oxidative stress have been reported to occur after exposure to HHP [19]. Transmission electron microscopy (TEM) and scanning electron microscopy (SEM) offer the possibility of identifying the cellular structures affected by HHP. For instance, some authors have described by TEM the presence of enlarged fibrillar regions and amorphous compacted regions, corresponding to denaturated DNA and cytoplasmic proteins. In addition, cells with a rougher surface and blister-like protrusions have been observed with SEM [20,21,22]. Fourier transform infrared (FTIR) spectroscopy is a vibrational spectroscopic technique that enables the biochemical signatures from microbiological structures to be extracted and analyzed and, therefore, has recently emerged as a useful methodology for the study of the mechanisms of sublethal injury and death induction resulting from bacterial exposure to food processing technologies, antimicrobial compounds and adverse environmental conditions [23,24]. Since FTIR spectra provide information on the biochemical composition of the main cellular constituents, the study of HHP-treated cells by FTIR spectroscopy may assist the identification of the cellular targets that result in being damaged after exposure to this food processing technology.

This study aimed to determine the morphological and physico-chemical changes occurring in cells of *E. coli* strains after HHP treatments of different intensities. For this purpose, the uptake of the fluorescent probe, propidium iodide (PI), and the loss of intracellular contents were used as indicators of membrane damage, while morphological alterations were analyzed by TEM and global changes in cellular biochemical features were assessed by FTIR spectroscopy.

## 2. Results and Discussion

### 2.1. Results

**Figure 1:**
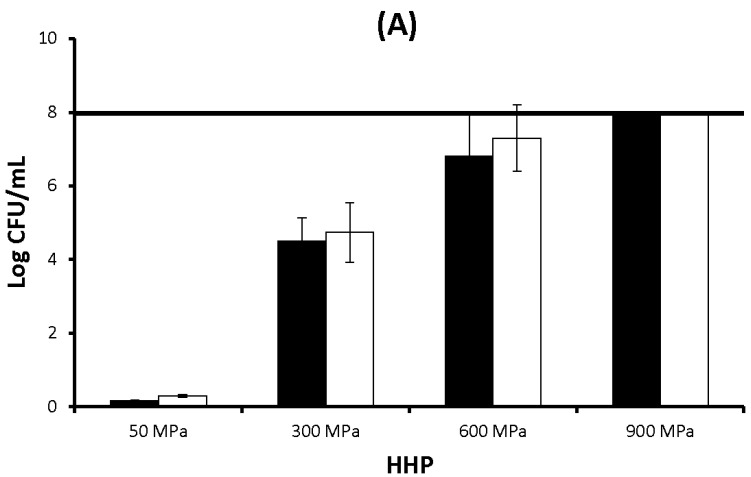

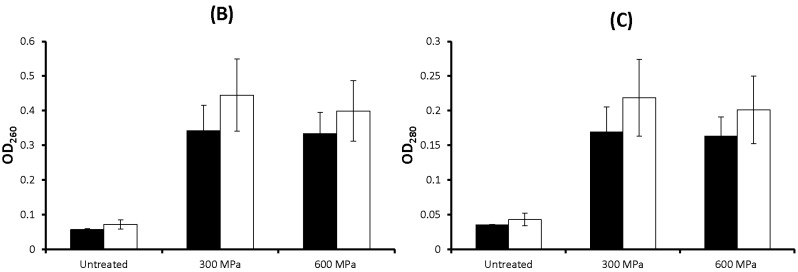
(**A**) Inactivation of *Escherichia coli* E218/02 (black bars) and *Escherichia coli* C-600 (white bars) by high hydrostatic pressure (HHP) (log CFU/mL); (**B**) OD_260_ of cell-free filtrates of *Escherichia coli* E218/02 (black bars) and *Escherichia coli* C-600 (white bars) untreated or treated with HHP at 300 MPa and 600 MPa; (**C**) OD_280_ of cell-free filtrates of *Escherichia coli* E218/02 (black bars) and *Escherichia coli* C-600 (white bars) untreated or treated with HHP at 300 MPa and 600 MPa. Results are shown as the average ± standard deviation.

Stationary-phase cultures of *Escherichia coli* E218/02 and *Escherichia coli* C-600 were exposed to different pressure-time combinations (50 MPa, 24 h; 300 MPa, 5 min; 600 MPa, 5 min; and 900 MPa, 5 min). Whereas HHP treatments at 50 MPa for 24 h did not give rise to significant reductions in the bacterial population, exposure to 300 MPa for 5 min resulted in the inactivation of 4.5 and 4.7 log cycles for *E. coli* E218/02 and *E. coli* C-600, respectively, and treatment at 600 MPa for 5 min gave rise to a 6.8 and 7.3 log reduction, respectively (Figure 1A). No survivors were found after HHP treatments at 900 MPa for 5 min (>8 log cycles of inactivation). No significant differences in HHP resistance were observed between both *E. coli* strains at the pressure-time combinations assayed.

The leakage of intracellular nucleic acids and proteins after HHP treatment at 300 MPa for 5 min and 600 MPa for 5 min was assessed by monitoring the optical density at 260 nm (OD_260_) and 280 nm (OD_280_) of cell-free filtrates from untreated and treated samples (Figure 1B,C). HHP treatment at 300 MPa caused a significant increase in both OD_260_ and OD_280_ of a similar magnitude for both *E. coli* strains. A further increase in pressure intensity (600 MPa) did not result in a further increase in OD_260_ and OD_280_ values, which were similar to those observed for cells treated at 300 MPa. No significant differences in OD_260_ and OD_280_ values were observed between *E. coli* E218/02 and *E. coli* C-600 at any of the treatment conditions.

**Figure 2:**
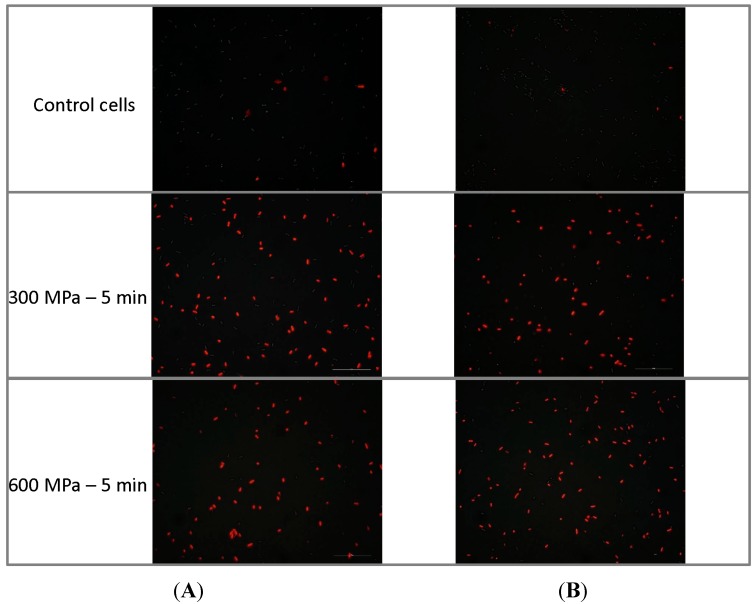
Uptake of propidium iodide by cells of *Escherichia coli* E218/02 (**A**) and *Escherichia coli* C-600 (**B**) untreated or treated with HHP at 300 MPa and 600 MPa followed by fluorescence microscopy.

Membrane integrity was also evaluated by following through fluorescence microscopy the intake of propidium iodide (PI) by untreated and HHP-treated populations (Figure 2). PI enters the cell when membrane integrity is compromised and binds to intracellular nucleic acids. Whereas less than 10% of untreated cells were stained when exposed to PI, the vast majority of HHP-treated cells (300 and 600 MPa for 5 min) were PI stained, which indicates the presence of HHP-induced damage in bacterial membranes.

**Figure 3:**
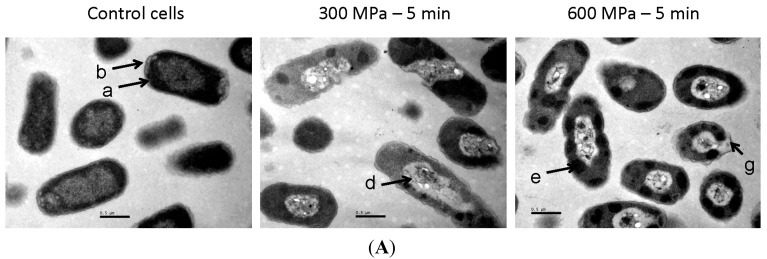

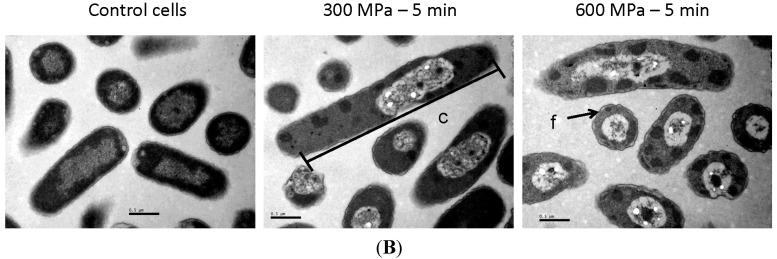
Representative electron micrograph sections of cells of *Escherichia coli* E218/02 (**A**) and *Escherichia coli* C-600 (**B**) untreated or treated with HHP at 300 MPa and 600 MPa. a: cytoplasmic membrane; b: cell wall; c: enlarged cell; d: disorganization of the genome area; e: condensation of cytoplasmic material in amorphous regions; f: membrane with winding shape; g: detached membrane.

The effects of HHP treatment on the ultrastructure of *E. coli* E218/02 and *E. coli* C-600 were assessed by using transmission electron microscopy (Figure 3). Untreated samples consisted mainly of single or dividing cells with a centrally-situated genome surrounded by the cytoplasmic area with tightly-packed ribosomes. Cell membranes and walls of these untreated cells were clearly distinguished. Cells within HHP-treated samples showed an altered appearance, with enlarged sizes and loss of the general cellular shape. Disorganization of the genome area was apparent, with the presence of blank spaces in the cytoplasm and condensation of the cytoplasmic material in amorphous compacted regions. Bacterial membranes presented winding shapes and sometimes were disrupted or detached from the cytoplasmic content. Similar ultrastructural alterations were observed for both *E. coli* strains at the two HHP treatment intensities (300 and 600 MPa for 5 min).

**Figure 4:**
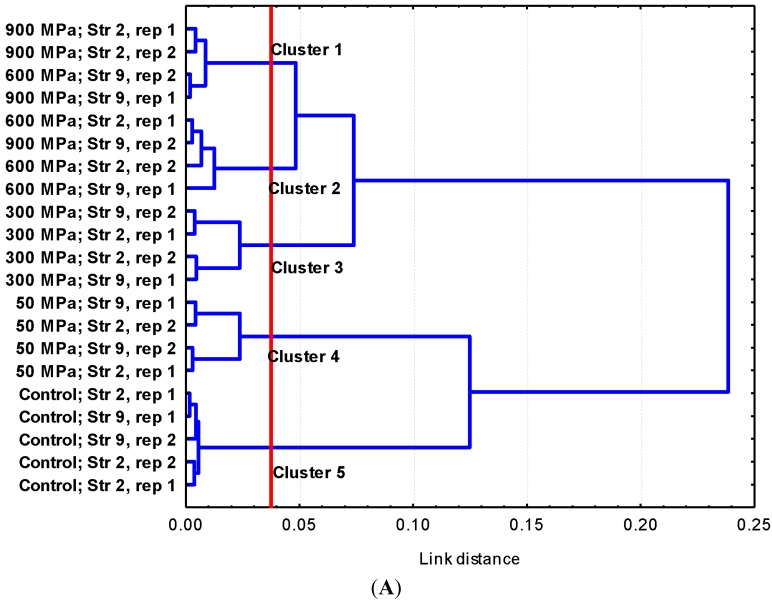

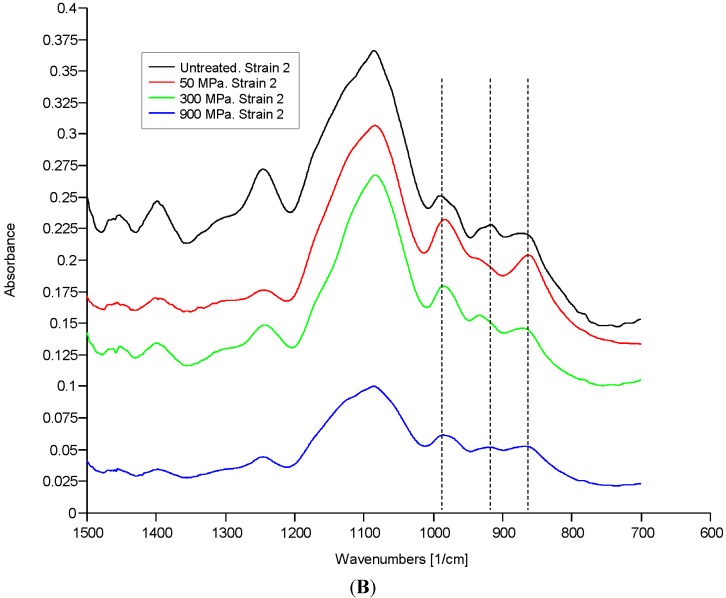
(**A**) Dendrogram obtained from w_4_ (1200 to 900 cm^−1^) spectral region data of two replicates (rep 1; rep 2) of strains *Escherichia coli* E218/02 (Strain number 2, Str 2) and *Escherichia coli* C-600 (Strain number 9, Str 9) untreated or HHP-treated cells, with cluster analysis performed with the Pearson product moment correlation coefficient and by the Ward algorithm method [25]; (**B**) untransformed FTIR spectra (w_3_, w_4_ and w_5_ spectral ranges,) of *E. coli* E218/02 (Str 2) before and after HHP treatment at different pressures (50, 300 and 900 MPa).

The changes in the molecular composition after HHP treatment were determined by using FTIR spectroscopy (Figure 4). Although no major modifications were visibly observed in the untransformed spectra, when the spectra were further processed, the chemically-based spectral differences were amplified and some minor modifications could be observed. Transformation of spectra included normalization, which balanced the differences in path strength, smoothing, which eliminated the instrumental noise, and second derivatization, which separated absorption bands, removed baseline shifts and increased spectral resolution. Measurement of the reproducibility of spectra by using the Pearson coefficient showed that low variability among replicates existed. Spectral windows w_1_ (0.97; 0.82) and w_4_ (3.21; 3.59) showed the lowest differentiation indexes (*DI*) and, consequently, the highest reproducibility, which demonstrates a good standardization of growth conditions and preparation of samples.

Hierarchical cluster analysis (HCA) of the second derivative spectra demonstrated that the w_4_ region was the most discriminant one. HCA of this spectral region divided the samples into two main groups separated with a linkage distance of 0.25, one including untreated samples (cluster 5) and samples treated at 50 MPa (cluster 4) and other comprising samples treated at higher pressures with lethal effects on bacterial cells (300 (cluster 3), 600 and 900 MPa (clusters 4 and 5)) (Figure 4). No significant differences were observed for other spectral regions between untreated and HHP-treated cultures.

### 2.2. Discussion

HHP represents a novel food processing technology that can be used as an alternative to conventional heat treatments in order to guarantee microbial inactivation and food safety, while causing minor effects on the quality and sensorial attributes of foodstuffs. The current study demonstrates the efficacy of HHP treatments to inactivate VTEC. Although recent investigations have focused on the elucidation of the mechanisms of bacterial inactivation and death by HHP [11,12,13,14], no definitive conclusions have been drawn yet. Our study evaluates how HHP treatments of varying intensity alter, to a different extent, the membrane integrity, ultrastructure and molecular composition of *E. coli* cells.

The ability of high pressures to damage the bacterial membrane was assessed by measuring the release of intracellular contents and the uptake of PI (dye that enters the cell when membrane integrity is compromised and binds to intracellular nucleic acids) following exposure to 300 and 600 MPa for 5 min. Treatment at both pressure intensities gave rise to a leakage of intracellular nucleic acids and proteins, as shown by the increase in OD_260_ and OD_280_ of cell-free filtrates. This leakage of intracellular components was of a similar magnitude at 300 and 600 MPa and suggests that HHP may cause disruption in the cellular envelopes. The vast majority of HHP-treated (300 and 600 MPa) cells were also stained after incubation with the dye PI, as shown by fluorescence microscopy observations. This fact evidences that their membrane was damaged, allowing for the uptake of this dye. Thus, membrane integrity tests showed that HHP treatments disrupt the cell envelopes, rendering them more permeable. These findings are in agreement with previous studies that demonstrated the occurrence of losses of physical integrity of outer and inner membranes after exposure to high pressures [11,12,13,14]. Monitoring of cellular ultrastructure allowed for the identification of alterations in the general cellular shape and structure. HHP treatment at 300 and 600 MPa gave rise to cellular enlargement, disorganization of the genome area, condensation of the cytoplasmic material in amorphous compacted regions and bending, disruption or detachment of cellular envelopes. These morphological alterations may be linked to denaturation and release of DNA and intracellular proteins. Ultrastructural modifications reported in the current study agree with previous observations by other authors through transmission electron microscopy (TEM) and scanning electron microscopy (SEM), describing the presence of enlarged fibrillar regions and amorphous compacted regions, corresponding to denaturated DNA and cytoplasmic proteins and identifying cells with a rougher surface and blister-like protrusions following exposure to high pressures [20,21,22]. FTIR spectroscopy has been recently proposed as a promising technique to study the mechanisms of sublethal injury and death induction by food processing methodologies and by exposure to antimicrobial compounds and adverse environmental conditions [23,26,27]. FTIR spectroscopic measurements showed that modifications in molecular compositions following HHP treatment were small and focused at the w_4_ spectral region (1200 to 900 cm^−1^, Figure 4B), which is mainly dominated by the ring vibrations of the functional groups C-O-C and C-O from the carbohydrates and polysaccharides of the cell wall and very specific, weak spectral patterns from ring vibrations of aromatic amino acids (tyrosine, tryptophan, phenylalanine) and nucleotides at the w_5_ spectral region (900–850 cm^−1^). Changes observed in these spectral regions suggest the presence of compositional or conformational alterations in some components of the external cell envelopes occurring in response to high pressures. Nonetheless, changes in FTIR spectra were minor in comparison to those previously described for *E. coli* cells exposed to a range of lethal stress conditions [9,28,29].

Ultrastructural observations, membrane integrity tests and FTIR spectroscopy analysis showed that structural and physico-chemical alterations caused by HHP treatments were not dependent on the pressure intensity of the lethal HHP treatment. *E. coli* treatment at 300 MPa gave rise to bacterial inactivation and significant structural and physico-chemical modifications. Treatment at 600 MPa caused a higher level of bacterial inactivation, but did not give rise to any additional structural/compositional alterations. This suggests that bacterial damage occurs once a threshold of pressure is reached and that different levels of damage recovery could happen as a function of the treatment intensity.

## 3. Experimental Section

### 3.1. Bacterial Strains and Culture Conditions

Two *E. coli* strains were used throughout this study, *Escherichia coli* E218/02, a VTEC O157:H7 strain originally isolated from a dry-fermented sausage involved in an outbreak in Sweden [30], and *Escherichia coli* C-600, a non-pathogenic laboratory strain. Cultures were maintained in cryovials at −80 °C. Bacteria were resuscitated in tubes containing 10 mL of brain heart infusion (BHI; Oxoid) by incubation at 37 °C for 24 h followed by streaking on BHI agar plates, which were then incubated under the same conditions. Precultures were prepared by transferring an isolated colony from a plate into a test tube containing 10 mL of sterile BHI followed by incubation at 37 °C for 24 h. Precultures were subsequently used to inoculate 50 mL of sterile BHI with approximately 10^3^ cells/mL, followed by incubation at 37 °C for 24 h, which resulted in a stationary phase culture with approximately 10^9^ cells/mL. This culture was subsequently used for HHP treatments.

### 3.2. HHP Treatments

Aliquots of 10 mL of the bacterial cultures were centrifuged at 8000× *g* for 5 min, and the cellular pellets were resuspended in 10 mL of PBS. Afterwards, suspensions (4 mL) were placed in heat-sealed sterile plastic pouches before pressurization. The pressure-time combinations tested were 50 MPa for 24 h, 300 MPa for 5 min, 600 MPa for 5 min and 900 MPa for 5 min. HHP treatments were performed in a Model FPG 7100:9/2C Series Foodlab (Standsted Fluid Power Ltd., Essex, UK). Pressurization water and the vessel were at 12 °C ± 1 °C before pressure treatment. The transmission fluid was a mixture of water and 30% propylene glycol. The adiabatic temperature increased 2–3 °C for every 100 MPa; thus, the maximum temperature range under 600 MPa and 900 MPa was 24–30 °C and 38–39 °C, respectively. The time taken to achieve the maximum holding pressure (900 MPa) was 50 s, and the decompression time was about two seconds. Pouches were removed immediately after treatment and aseptically opened. Ten-fold serial dilutions were produced in sterile 0.1% (*w*/*v*) peptone solution; suitable dilutions were plated on BHI agar, and viable cells were enumerated following the incubation of plates at 37 °C for 48 h (longer incubation times did not show any influence on the count).

### 3.3. Membrane Integrity Tests

#### 3.3.1. Assessment of Propidium Iodide (PI) Uptake

Control untreated cultures and bacterial cultures exposed to the different pressure-time regimes were subsequently diluted in PBS to achieve a final cellular concentration of ~10^8^ cells/mL. Afterwards, 1 µL of PI (Molecular Probes, Life Technologies, Grand Island, NY, USA) was added, and the mix was incubated in the dark at room temperature for 10 min. Finally, the cell suspension was centrifuged; the cellular pellet was suspended in PBS, and samples were analyzed by Epi-fluorescence microscopy (Model E 600, Nikon, Tokio, Japan) with a high-pressure mercury lamp (Model HB-10104AF, Nikon) and filter cube (or optical block) blue excitation filter combination (Model B-2A, Nikon) with spectral profiles as follows: Excitation (EX) filter 450–490 nm, Dichroic Mirror (DM) filter 505 nm, Barrier (BA) filter 520 nm. The cells that stained red were considered as non-viable.

#### 3.3.2. Measurement of Cellular Leakage

Aliquots of 3 mL of cell cultures exposed to different pressure-time regimes were filtered through a 25-mm-diameter, 0.22-µm pore size Millex-GS syringe filter (Millex-GS, Millipore Co, Billerica, MA, USA), and the presence of nucleic acids and proteins in the cell-free filtrate was checked by measuring the absorbance at 260 nm and 280 nm, respectively (Beckman DU 7400 Spectrophotometer, Beckman Coulter, Inc., Brea, CA, USA), as described elsewhere [26].

### 3.4. Transmission Electron Microscopy (TEM) Analyses

*E. coli* cells exposed to 300 and 600 MPa for 5 min were harvested by centrifugation and fixed in 2.5% glutaraldehyde (TAAB Laboratories Ltd., Aldermaston, Berks, UK)-PBS for 3 h at 4 °C. Bacteria were then washed three times with PBS, and cells were treated with osmium tetroxide (TAAB Laboratories)-1% PBS for 45 min at room temperature in darkness. Afterwards, three new washings with PBS were performed. Subsequently, cells were pelleted in bacteriological agar (Oxoid, Hampshire, UK), and pellets were dehydrated in ethanol solutions of increasing concentrations and embedded in an epoxy resin (Epon 812; Tousimis, Rockville, MD, USA), which was polarized by its incubation for 48 h at 60 °C. Finally, ultrathin sections were collected onto copper grids and stained with uranyl and lead. Microscopic observations were carried out on at least ten different microscope fields per treatment condition, using several microscope magnifications of a JEOL 1010 microscope (JEOL Ltd., Tokio, Japan) at 80 kV [26].

### 3.5. Fourier Transform Infrared (FTIR) Spectroscopic Analyses

Control cells and cells exposed to 50, 300, 600 and 900 MPa were harvested by centrifugation and suspended in 50 µL of PBS, placed (15 µL) in a ZnSe window and stove dried (15 min, 50 °C). Infrared spectra were obtained with a FTIR spectroscope (Perkin-Elmer 2000 FTIR, Massachusetts, MA, USA) equipped with a Deuterated Triglycerine Sulfate (DTGS) detector. Measurements were recorded over the wavelength range of 4000 to 700 cm^−1^ with an interval of 1 cm^−1^. The spectral resolution was 4 cm^−1^. The final spectra were achieved averaging 20 scans. FTIR experiments were performed in triplicate. A software application developed for the Perkin-Elmer environment was used for transformation, including normalization (0 setting with absorption at 1800 cm^−1^; 1 setting at maximal absorption, located around 1650 cm^−1^), smoothing and second derivative. After transformation, spectra were recorded in ASCII format and processed [31]. The whole spectrum was divided for calculation purposes into five spectral windows: the window between 3000 and 2800 cm^−1^, influenced by functional groups of membrane fatty acids (w_1_); the window between 1800 and 1500 cm^−1^, affected by amide I and amide II groups belonging to proteins and peptides (w_2_); the window between 1500 and 1200 cm^−1^, mixed region influenced by proteins, fatty acids and phosphate-carrying compounds (w_3_); the window between 1200 and 900 cm^−1^, which is informative mostly for the carbohydrates and polysaccharides in the cell wall (w_4_); and the window between 900 and 700 cm^−1^, named the true fingerprint, because of very specific spectral patterns (w_5_).

To study variability between replicates and within windows, samples were processed, in independent experiments, yielding three replicates for each HHP treatment condition. The differentiation index (*DI*) [32] was calculated for each pair of the IR spectra, for the working IR range (3000–2800; 1800–700 cm^−1^) and independently for the ranges described (w_1_ − w_5_), according to the equations.

(1) $$DI_{y\text{1}y\text{2}}=\;\left(\text{1 }-r_{y\text{1}y\text{2}}\right)\;\times \text{ 1}000$$

(2) $$r_{y\text{1}y\text{2}}=\frac{\sum_{i=1}^{n}y1_{i}y2_{i}-n\bar{y1}\bar{y2}}{\sqrt{\sum_{i=1}^{n}y1_{i}^{2}-n{\bar{y1}}^{2}}\sqrt{\sum_{i=1}^{n}y2_{i}^{2}-n{\bar{y2}}^{2}}}$$

where *r*_y1y2_ is the Pearson’s correlation coefficient, *y*1_i_ and *y*2_i_ are the individual absorbance values of the two spectra to be compared, *n* is the number of data points in the given range and $\bar{y1}$ and $\bar{y2}$ are the arithmetic mean values of *y*1 and *y*2.

The three replicates for each combination (1–2; 1–3; 1–3) are considered and the mean is obtained. *DI* may adopt values between zero and 2,000, with zero for identical spectra, 1000 for completely non-correlated and 2000 for completely negatively non-correlated spectra [32].

The spectral data were subjected to multivariate statistical methods (Hierarchical Cluster Analysis (HCA) and Factor Analysis (FA)). Pearson’s product moment correlation coefficient was used to measure the similarity between spectra, and strain clustering was achieved using Ward’s algorithm. All of the analyses (calculation of coefficients, joining of variables, canonical analysis and graphical display) were carried out with the Statistica for Windows, v. 7.0, program (Statsoft Inc., Tulsa, OK, USA).

## 4. Conclusions

Our findings evidence that while exposure of *E. coli* cells to HHP causes alterations in their physical integrity, with cellular enlargement, membrane damage or detachment, DNA and protein denaturation and leakage of intracellular contents, minor changes in molecular composition are evident, and these are focused in the carbohydrates and polysaccharides of the cell wall. These observations serve to enhance the currently-available knowledge on the mechanisms of bacterial inactivation by HHP and may be valuable to design more effective food preservation regimes based on the integration of mild HHP into a multi-target hurdle technology concept. For instance, our results show that HHP compromises membrane integrity, which suggests that mild HHP might increase the efficacy of food-grade antimicrobials, such as nisin, or other natural antimicrobials that otherwise might be unable to enter the cell.
